# A metacontinuum model for phase gradient metasurfaces

**DOI:** 10.1038/s41598-023-39956-z

**Published:** 2023-08-10

**Authors:** Giorgio Palma, Umberto Iemma

**Affiliations:** Department of Civil, Computer Science and Aeronautical Technologies Engineering, Roma Tre University, 00146 Rome, Italy

**Keywords:** Acoustics, Computational methods

## Abstract

Acoustic metamaterials and metasurfaces often present complex geometries and microstructures. The development of models of reduced complexity is fundamental to alleviate the computational cost of their analysis and derivation of optimal designs. The main objective of this paper is the derivation and validation of a metacontinuum model for phase gradient-based metasurfaces. The method is based on the transformation acoustics framework and defines the metasurface in terms of anisotropic inertia and bulk modulus. Thermal and viscous dissipation effects in the metacontinuum are accounted for by introducing a complex-valued speed of sound. The model is implemented in a commercial FEM code, and its predictions are compared with numerical simulations on the original geometry and also using an equivalent boundary impedance approach. The results are examined for an exterior acoustics benchmark and for an in-duct installation in terms of transmission coefficient with the four-pole matrix method. The metacontinuum model gives solid results for the prediction of the acoustic properties of the examined metasurface samples for all the analyzed configurations, as accurate as the equivalent impedance model on which it is based and outperforming it in some circumstances.

## Introduction

The idea of metamaterials was initially developed in electromagnetism with the first definitions of the term, describing them as “three-dimensional, periodic cellular architectures designed to produce an optimized combination not available in nature of two or more responses to a specific excitation”^1^. Cui et al.^2^ added in their definition that the response of a metamaterial is due “both to the cellular architecture and chemical composition” of the periodic or non-periodic structure. Some review articles have been published in the last decade, dealing with acoustics^3–5^ and aeroacoustics^6^, collecting many different concepts. The term metamaterial has often been connected to resonant behaviours, typically manifesting in specific frequency ranges connected to the periodic resonant unit’s sizes (usually of subwavelength scale)^7^. A subtle aspect of these definitions is the risk of rebranding old concepts with a new and fancier name. The etymology of the term “meta”, from the Greek $\mu \epsilon \tau \acute{\alpha }$, meaning “after” or “beyond”, offers the possibility to define metamaterials as a “more comprehensive” class of materials, or, equivalently, materials “transcending” the classic ones in terms of properties. It can be stated that a metamaterial requires extended modelling at the macro scale level to capture its response, going beyond the standard accurately and well-established governing laws of the subject(s) involved. It is the case, for example, of concepts showing equivalent negative inertia or bulk modulus^8,9^, or metafluids^10^ and metacontinua^11^ in acoustics, which include anisotropic inertia and bulk modulus into the governing equations allowing the effective modelling of behaviours such as acoustic cloaking^11–18^. Recently, bianisotropy in acoustics, or Willis coupling, has been proven and investigated, opening the way to transpose effects enabled by bianisotropy from electrodynamics to acoustics^19,20^. Furthermore, recent advances have shown that, by locally controlling the bianisotropic response of the cells, one can ensure full control of refraction in gradient metasurfaces, avoiding scattering towards unwanted directions^21^, with unitary efficiency^20^. The analytical modeling based on Willis coupling showed the capability to derive the parameters, describing the scatterer of a metasurface, tailored to achieve perfect reflection, asymmetric reflection and absorption^22^. It is worth noting that even some old and well-known concepts may be effectively studied using new and extended metamaterial models, which can better expose some of their peculiarities.

Acoustic phase-gradient metasurfaces (PGMs) have been developed as a way for manipulating the acoustic refraction or reflection from boundaries in terms of direction, wavefronts’ shape, and wave amplitude modulation^23^, through tailored arrangements of phase delays introduced in the acoustic field compared to the untreated case. Typically (PGMs) are designed involving repetitions, possibly periodic, of elementary cells. The $2\pi$ phase delay range is usually sampled in an integer number of discrete steps, defining the number *n* of different elementary cells, each one designed to provide one delay level in the reflected or transmitted acoustic field. The phase delay distribution on the treated boundary can be effectively shaped by properly arranging the elementary cells on the boundary. The Generalized Snell’s Law constitutes the connection between the desired metabehaviour from the PGM and the required phase delay gradient profile over the boundary. The concept of phase manipulating metasurfaces has been first introduced in electromagnetism^24^, where it is also exploited to control reflection and diffraction from treated boundaries, and transmission through metasurfaces, also manipulating the dispersion of optical systems^25^. Recently, Li et al.^26^ demonstrated the possibility to decouple and independently manipulate two co-polarized transmission components introducing a chirality-assisted phase, that impose separate phase profiles in two circular polarization preserving channels. In the acoustic field, carefully designing the metasurface allows obtaining exotic effects such as extraordinary (non-geometric) reflection angles from boundaries, focusing of acoustic waves^27^, one-way acoustic transmission^28^, dynamic wave control and smart materials^29,30^, the transformation of wavefront shapes from plane to spherical, mode conversion into surface waves^31^, carpet cloaking^32^, acoustic holograms^33^, and acoustic illusions^34^. The unit cells are generally designed to form a locally reacting metasurface, in the sense that rigid boundaries physically separate the cells. However, some exceptions can be found in the literature^35^ that exploit non-locality to enhance the device’s performance. Thin devices with broadband effect^36,37^, also achieved with design by optimization^38,39^, received particular attention. Results have been obtained in the literature with cells of various shapes, such as straight tubes^37,40^, space-coiling (SC) cells^23,41,42^, spiral structures^31,36,43^, Helmholtz resonators (HR)^44,45^, or more complex microstructures^46,47^, modelling their effects using several approaches, such as equivalent refractive index^48^, equivalent impedance^23^, effective isotropic medium with equivalent density and speed of sound^46^. One of the characteristics of metamaterials and metasurfaces is that their structure can be very complex also at a micro-level. This fact may cause the acoustic numerical simulations of their complete geometry to become very expensive, needing to include all the small features of the design in the discretized domain. For this reason, the availability of simplified and reliable models is a key aspect of the simulation of their acoustic behaviour and their design optimization.

In this article, we derive a general equivalent metacontinuum model that represents the metasurface cells in terms of equivalent (possibly anisotropic) bulk modulus and inertia tensor, starting from the geometry of the cell. The properties of the metacontinuum are linked to the phase delay introduced by the cell, stating the connection with the Generalized Snell Law. The resulting model considers the cell as a continuum, strongly reducing the complexity of the domain to be simulated. The paper deals in particular with Helmholtz resonator and space-coiling cells; nonetheless, the model is valid in principle for other cell designs, as extending it to other concepts exploiting the parallelism with acoustic impedance is easy. Numerical simulations confirm the validity of the metacontinuum model for modeling PGMs. The method is generally based on the equivalent impedance model and hence shares the same level of accuracy in the numerical simulations of the cells. Incidentally, it is found that, in some particular circumstances, the simplifications introduced by the impedance model produce some numerical issues, which are avoided by the metacontinuum. The metacontinuum model can also be seen as a hint on how cells for PGMs can be actually built. The strong connection of the metacontinuum with pentamode materials suggests that periodic lattices with tailored microstructure can be exploited to obtain the possibly anisotropic properties of the continuum needed. The use of such structures would allow for combining the acoustic response with peculiar mechanical properties for the cells.

## Results

### The metacontinuum formulation

An acoustic metafluid, a particular case of metacontinuum, is a metamaterial that behaves acoustically as a fluid due to the null, or near-zero in practical realizations^49^, shear modulus exhibited by its structure. The propagation of an acoustic disturbance within the most general metafluid is effectively described by^10,11^

1 $$\begin{aligned} - {\frac{\partial ^2 {p}}{\partial {t}^2}} + {c_{\tiny {\textsf {ref}}}^2}\;\hat{K} \; \nabla \cdot \left( \textbf{Q}\,\varvec{\hat{\rho }}^{-1}\,\textbf{Q}\;\nabla p \right) = 0 \end{aligned}$$

where $\varvec{\rho }=\varvec{\hat{\rho }}\varvec{\rho }_{\tiny {\textsf {ref}}}$ represents the anisotropic inertia of the material, $K = \hat{K}K_{\textrm{ref}}$, $\textbf{Q}$ can be any symmetric tensor such that $\nabla \cdot \textbf{Q}=0$ and the Cauchy stress tensor for such a material is given by $\varvec{\sigma }=-p\,\textbf{Q}$; $\varvec{\rho }_{\tiny {\textsf {ref}}}$, $K_{\textrm{ref}}$, and $c_{\tiny {\textsf {ref}}}=\sqrt{K_{\textrm{ref}}/\varvec{\rho }_{\tiny {\textsf {ref}}}}$ are the reference density, bulk modulus, and speed of sound, respectively, which may be taken equal to the values for the hosting fluid, without loss of generality.

#### Space-coiling cells

**Figure 1:**
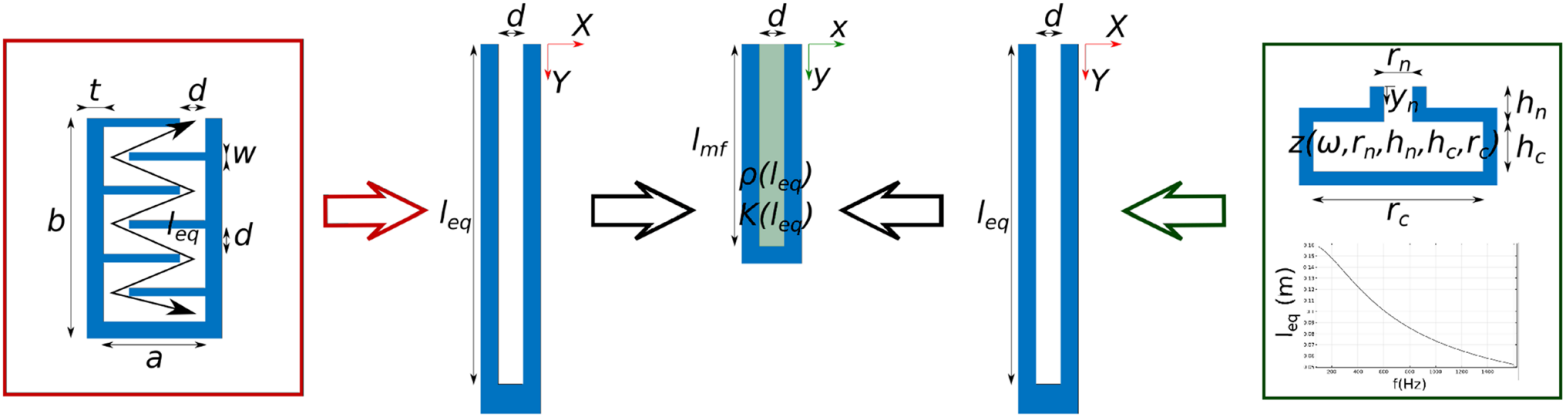
The metacontinuum properties are obtained from the knowledge of the cell design. For the space-coiling cell, first the equivalent length $l_{\text {eq}}$ is evaluated from the path of the wave propagating in the cell channels, following Eq. (3); this is then converted in the metacontinuum properties $\varvec{\rho }$ and *K* following Eq. (4). The case of the Helmholtz resonator cell defines the more general approach, in which the equivalent length is identified using the equivalent impedance as a preliminary step.. In both cases, $l_{\text {mf}}$ is arbitrarily chosen, *e.g.* equal to the original thickness of the cells.

We start deriving the equivalent metacontinuum for a general space-coiling cell, which is a modification of the quarter-wavelength resonator concept, in which the channel is folded multiple times to reduce the device’s overall thickness. The design sketch with its sizing parameters is shown in Fig. 1. The cell is characterized by its depth *b* and overall width $a+2t$, being t the thickness of the external walls; the channels have a constant cross-section *d*, with the channels separated by internal septa of thickness *w* and width $l_w$. Typically, the channels have subwavelength width, being waveguides for the 0th-order mode. There always exists for a coiled cell an equivalent straight channel length, $l_{\text {eq}} = g(l)$, which is a function of (but may differ from) the purely geometric evaluation of the unfolded channel extension. In Ghaffarivardavagh et al.^23^, a simple calculation of equivalent lengths for zig-zag cells is proposed, considering the diagonal path across the folded channels

2 $$\begin{aligned} l = \sqrt{(d+w)^2+a^2} \end{aligned}$$

Here, the reference value is modified by introducing linear correction depending on some cell design parameters (expressed in meters)

3 $$\begin{aligned}{}&l_{\text {eq}} = {\left\{ \begin{array}{ll} l + \frac{c_1(d-c_2)}{c_2} + \frac{c_3(a-c_4)}{c_4} + \frac{c_3(w-c_5)}{c_5} &{}d<0.009\\ l + \frac{c_1(d-c_2)}{c_2} + \frac{c_3(a-c_4)}{c_4} + \frac{c_3(w-c_5)}{c_5} + \frac{c_6 (d-c_7)}{c_7} &{}d>0.009, \end{array}\right. }\\&\text {with} \quad \begin{array}{c | c | c | c | c | c | c} c_1 &{} c_2 &{} c_3 &{} c_4 &{} c_5 &{} c_6 &{} c_7\\ \hline -0.0015 &{} 0.002 &{} 0.0009 &{} 0.022 &{} 0.001 &{} 0.0035 &{} 0.009 \end{array} \end{aligned}$$

The equivalent length is the fundamental parameter of such cells, directly defining the expected phase delay introduced in the reflected field compared to a flat boundary $\Delta \Phi = \frac{4 \pi l_{\text {eq}}}{\lambda _0}$ (fundamental for PGM applications) or, equivalently, the effective relative refractive index $n_{\textrm{eff}} = l_{\text {eq}}/t$ (with *t* the thickness of the metadevice), and also defining their equivalent specific surface acoustic impedance $z = -i \cot {(\frac{\omega }{c} l_{\text {eq}})}$, where $z =Z/(\rho c)$ and $Z=p/(\textbf{v}\cdot \textbf{n})$ is the acoustic impedance.

We can set a metafluid domain of arbitrary thickness $l_{\text {mf}}$, *e.g.* the thickness of the original cell, and, following the Transformation Acoustics method^10^, obtain an expression for the inertia tensor, bulk modulus, and $\textbf{Q}$ such that the metafluid cell will mimic the acoustic behavior of the original cell. To do that, a coordinate transformation is set between the original domain $\Omega$ and the metafluid $\omega$, through an invertible mapping $\Omega \rightarrow \omega$ defined by $\varvec{\xi }=\chi \varvec{(\Xi )}$, being the components of the transformation gradient ${\textbf{F}}$ described as $f_{ij} = \partial \xi _i / \partial \Xi _j$, $\varvec{\xi }$ = (*x*, *y*, *z*) and $\varvec{\Xi }$ = (*X*, *Y*, *Z*). Mapping the equivalent length in the metafluid thickness we obtain, see Fig. 1

$$\begin{aligned} x = X , \quad \quad y-y_0 = \frac{l_{\text {mf}}}{l_{\text {eq}}} (Y-Y_0), \quad \quad z = Z \quad \quad \textrm{with} \quad Y_0<Y<l_{\text {eq}}, \end{aligned}$$

The coordinate transformation set compresses the space in one direction only, *i.e.,* along the thickness of the cell. The width of the metacontinuum cell is defined to be equal to the width *d* of the channel of the original cell. This is imposed to keep unaltered the size of the interface between the cell and the hosting domain and maintain the equivalence between the original cell and the metacontinuum. In the following, the problem is addressed under the simplifying hypothesis of inertial metafluid, a special case of the more general class of metacontinua that implies ${\textbf{Q}}={\textbf{I}}$. In this case, the metacontinuum parameters are related to $\textbf{F}$ by

4 $$\begin{aligned} \hat{K} = \det \textbf{F}, \quad \varvec{\hat{\rho }}= \det (\textbf{F}) (\textbf{V}\textbf{V}^\text{T})^{-1} \end{aligned}$$

The corresponding deformation gradient ${\textbf{F}}$ is diagonal with components $f_{11} = 1, \; f_{22} = {l_{ms}}/{l_{\text {eq}}},$ and $f_{33} = 1$. Hence, the metafluid is characterized by

5 $$\begin{aligned} \begin{aligned} \hat{K} = f_{22}, \quad \varvec{\hat{\rho }}= f_{22} \begin{bmatrix} 1 &{} 0 &{} 0\\ 0 &{} \frac{1}{f_{22}^2} &{} 0\\ 0 &{} 0 &{} 1 \end{bmatrix} \end{aligned} \end{aligned}$$

It shall be noted that the assumption of inertial metafluid does not limit the analysis’s generality. As demonstrated by Norris^10^, the transformation presented belongs to a particular class that would allow transferring the anisotropicity entirely to the tensor $\textbf{Q}= \det ({\textbf{F}})^{-1} {\textbf{V}}$ (and hence to the stress tensor $\varvec{\sigma }$), removing the hypothesis of inertial metafluid. In this case, one obtains a pentamode material with isotropic inertia and anisotropic elasticity, which is typically easier to achieve than anisotropic inertia. For combinations of frequencies of the incoming acoustic perturbation and channels width such that only plane waves can propagate inside the channels, the complete metafluid model can be approximated neglecting the anisotropy arising from the application of the transformation acoustic theory. This is the case for example of the space-coiling cells studied in the Numerical assessment section, for which one can use the isotropic values $\hat{K}= f_{22}$ and $\hat{\rho }= 1/f_{22}$. However, since the modeling presented in this work is intended to be general, the complete formulation is used in the derivation of the numerical results. Anisotropic properties are needed, for example, when dealing with annular cells mounted on a duct wall with an uninterrupted annular interface with the hosting domain, which are azimuthally non locally reacting, and in general in all those situations where the cell is not a simple waveguide for plane waves, allowing two- and three-dimensional propagation of acoustic waves inside their domain.

#### From impedance to metacontinuum

The space coiling cell example is a particular case of a more general approach. When dealing with original concepts different from the quarter-wavelength resonator, an equivalent length can also be derived. This can be obtained by exploiting the well-known relation between the length and the acoustic specific impedance of a hard-backed straight cavity $z(\omega ) = -i \cot {(\frac{\omega }{c} l)}$, which can be inverted as

6 $$\begin{aligned} l_{\text {eq}} = \frac{c}{\omega } \cot ^{-1}\left( {\text {Im}\left( z\left( \omega \right) \right) }\right) \end{aligned}$$

For example, a metafluid model for a Helmholtz resonator can be derived starting from the definition of its equivalent surface impedance, which can be modelled using a lumped mechanical element equivalence^50^,

7 $$\begin{aligned}&z = \frac{1}{i\omega } (-\omega ^2 \rho _0 \hat{h}_{n} + \frac{c_{\tiny {\text { 0}}}^2 \rho _0 r_{n}}{h_{c} r_{c}}) \end{aligned}$$

Equation 7 comes from the equation of motion of a mass-spring system representing the resonator, and its derivation is reported in the Supplementary Information. The term $\hat{h}_{n}$ is the length of the neck of the resonator, accounting for the end corrections for each side of the neck^51,52^

8 $$\begin{aligned} \hat{h}_{n} = h_{n} + 2\left( 0.85 r_{n} \left( 1-1.33 \frac{r_{n}}{r_{c}}\right) \right) \end{aligned}$$

The equivalent length resulting from Eq. (6) will typically be frequency dependent; the case for the Helmholtz resonator cell is no exception, and its equivalent metafluid properties are then obtained by combining Eqs. ( 5, 6 and 7). The same considerations made in the Space-coiling cells section, on the use of an approximated isotropic model, hold for the general case as well, with the 1D-mirage approximation being valid until the zeroth order mode is the only one propagating in the equivalent straight channel of width *d*. Again, the model presented is intended to be generally applicable, and, for this reason, it has been derived using the complete anisotropic formulation that is used to obtain the results in the Numerical assessment section.

#### Thermoviscous losses

The metacontinuum model obtained so far neglects thermoviscous effects and losses that may happen in the original cell. These may be relevant and substantially modify the acoustic behavior of the modeled cells, especially when the design includes relatively narrow channels. Nevertheless, this type of lossy behavior can be easily included using a complex propagation constant and speed of sound. Following Pierce^53^, when the considered channel is large enough for the boundary layers to occupy a small fraction of its cross-section, the dispersion relation can be approximated by

9 $$\begin{aligned} k&= \frac{\omega }{c_{\tiny {\textsf {ref}}}} = \frac{\omega }{c_{\tiny {\text { 0}}}} + (1-i) a_{\text {walls}}, \quad \textrm{with} \quad a_{\text {walls}} = \frac{1}{c_{\tiny {\text { 0}}}d_{\text {eq}}} \sqrt{\frac{\omega \eta }{ 2 \rho _0 }} \left( 1 + \frac{\gamma -1}{\sqrt{Pr}} \right) \end{aligned}$$

with $\eta$, $d_{\text {eq}}$, $\gamma$, and *Pr* being the dynamic viscosity of the reference fluid, the equivalent diameter of the channel, the ratio of the specific heats, and the Prandtl number, respectively. Then, solving Eq. (9) for $c_{\tiny {\textsf {ref}}}$, the desired complex-valued speed of sound is obtained

10 $$\begin{aligned} c_{\tiny {\textsf {ref}}}= \left( \frac{1}{c_{\tiny {\text { 0}}}} + \frac{(1-i) a_{\textrm{wall}}}{\omega }\right) ^{-1} \end{aligned}$$

The Pierce model can be applied straightforwardly for designs involving a uniform cross-section, such as the quarter-wavelength resonator, using the channel width as the equivalent diameter. However, cells like the Helmholtz resonator present abrupt changes in the channel width between the neck and the cavity. In such cases, the original formulation involves using a different complex-valued propagation constant *k* for each part of the structure with different width. The metafluid domain should hence be partitioned recognizing the different parts of the structure, to use a different complex-valued speed of sound in each subdomain. In general, however, it would be difficult to map the original structure in the metafluid domain and hence define the correct partitioning, especially when the latter is generated starting from the equivalent impedance, see Eq. (6), even for simple structures. It is proposed here to use an equivalent value ${\hat{a}}_{\text {walls}}$ for the evaluation of the imaginary part of the speed of sound, evaluated as the average of the $a_{\text {walls},i}$, weighted with the relative extension $l_i$ of each part of the cell having different diameters

11 $$\begin{aligned} {\hat{a}}_{\text {walls}} = \sum _i l_i \, a_{\text {walls},i}(d_{\text {eq},i}), \quad \text {with } \sum _i l_i = 1 \end{aligned}$$

### Numerical assessment

**Figure 2:**
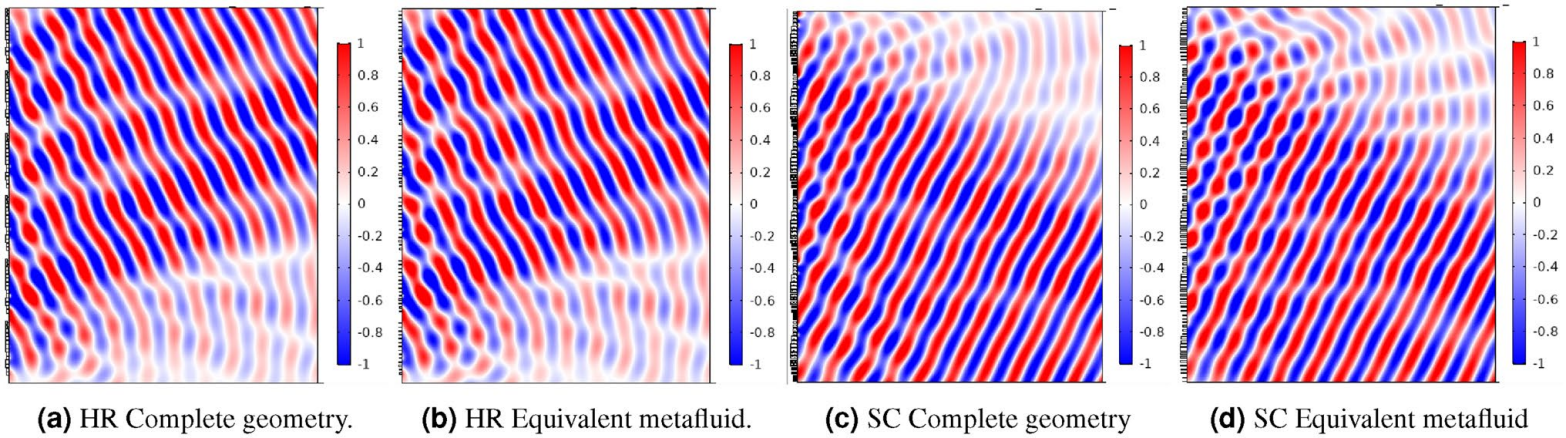
PGMs acoustic pressure fields: with Helmholtz resonator cells in (**a**) and (**b**) ($kw_{\text {cell}}=6.81$) and with space-coiling cells in (**c**) and (**d**) ($kw_{\text {cell}}=7.01$). The simulation involving the complete geometries (**a**) and (**c**) are compared to the results from the metafluid model (**b**) and (**d**).

The extraordinary reflection from a PGM is a benchmark commonly found in the literature for assessing acoustic metasurfaces performances. It is used here to qualitatively show the equivalence between the complete geometry and the metacontinuum model. The metasurface phase shift gradient is designed to achieve an additional reflection angle of $30^\circ$ accordingly to the Generalized Snell’s Law^42^. In Fig. 2a and b, lossless simulations are compared using a Helmholtz Resonators-based PGM, which geometric parameters are listed in the Supplementary Information. The agreement between the two simulations is excellent, as the pressure fields shown in the two figures are almost indistinguishable. The metasurface in Fig. 2c is built using SC cells and designed with the same criterion mentioned above (the list of the constructive parameters of the cells can be found in the Supplementary Information). The acoustic field presents minor differences when the metafluid model is used instead of the complete geometry, Fig. 2d. However, the simulations still capture the overall behaviour of the metasurface. The level of agreement achievable using the metafluid model depends primarily on the quality of the evaluation of the equivalent length (or impedance in the general case) for the cells. Hence, the two models share the same order of accuracy.

**Figure 3:**
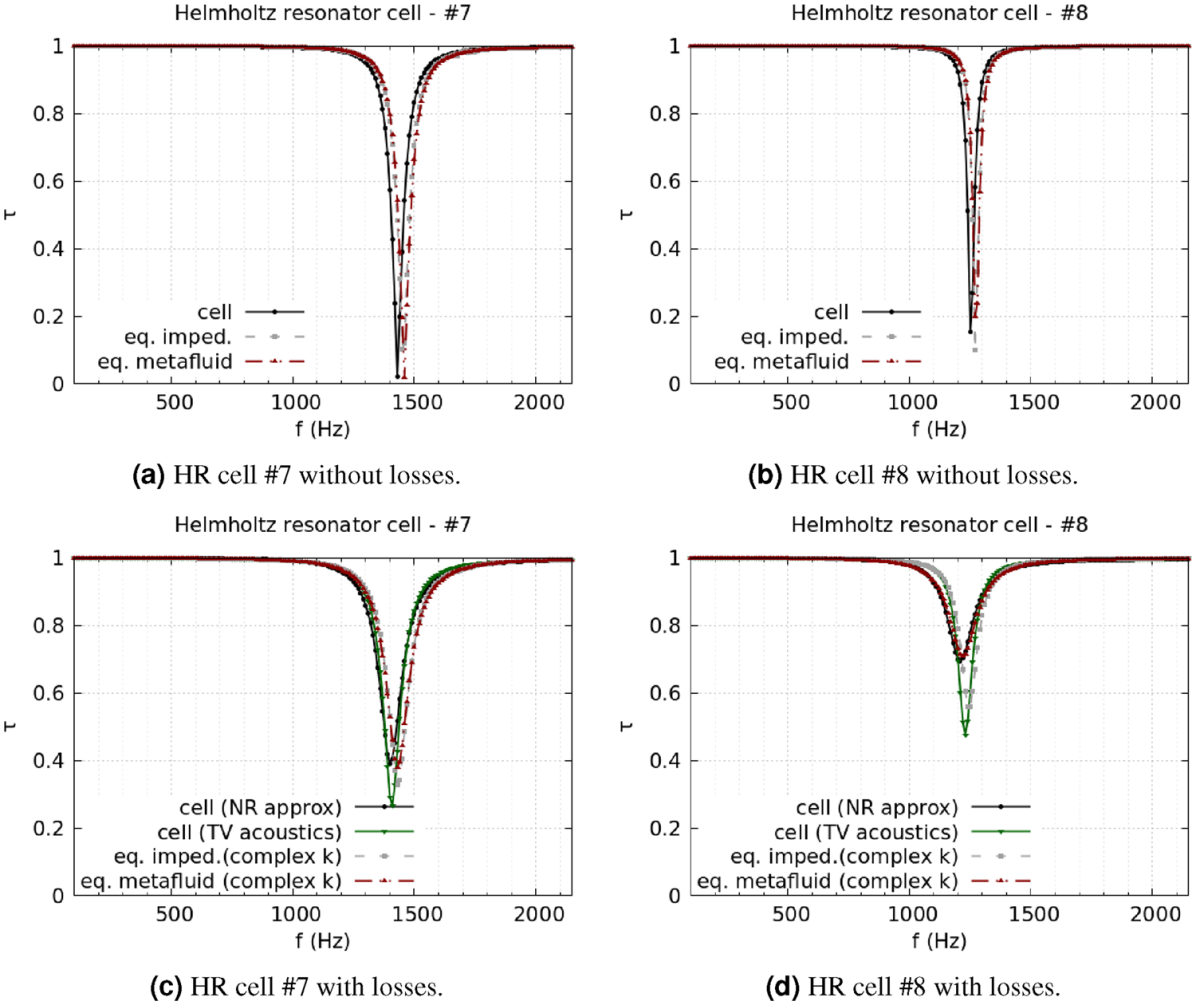
HR cells, comparison of transmission coefficient spectra between full geometry, equivalent impedance, and equivalent metafluid lossless simulations [(**a**) and (**b**) for cell #7 and #8, respectively]. For the lossy case [(**c**) and (**d**) for cell #7 and #8, respectively], a narrow regions approximation and a full thermoviscous acoustic simulation are compared with the equivalent impedance and metafluid modelling with complex wavenumber.

**Figure 4:**
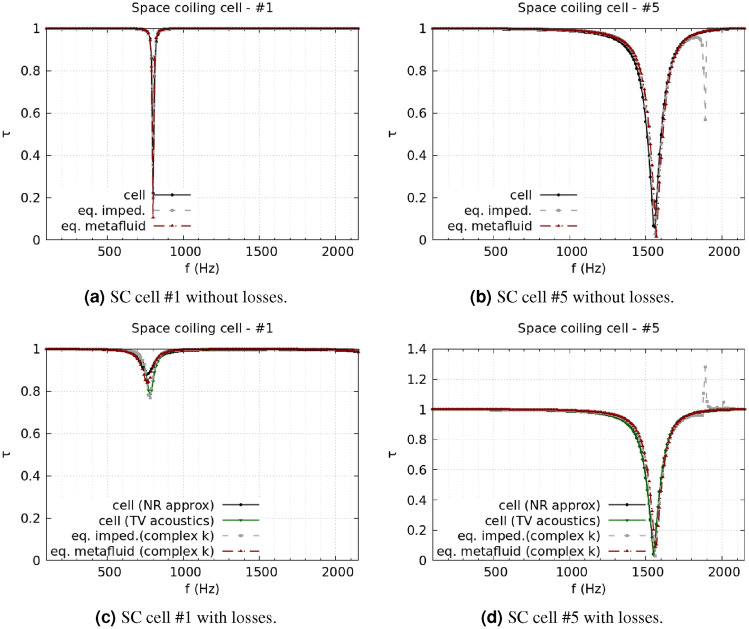
SC cells, comparison of transmission coefficient spectra between full geometry, equivalent impedance, and equivalent metafluid lossless simulations [(**a**) and (**b**) for cell #1 and #5, respectively]. For the lossy case [(**c**) and (**d**) for cell #1 and #5, respectively], narrow regions approximation (NR approx) and a full thermoviscous acoustic simulation (TV acoustics) are compared with the equivalent impedance and metafluid modelling with complex wavenumber (complex k).

The analysis of the duct transmission benchmark confirms the statement. Cells #7 and #8 are taken as the most representative of the HR design, and Fig. 3 shows the corresponding results. Figure 3a and b report the comparison between lossless simulations using the cells’ complete geometry, the impedance model, and the metafluid model. When no losses are considered, the impedance model and the metafluid model are expected to be equivalent, as the metafluid parameters are fully defined starting from the impedance. The same good accuracy level is indeed reached by the equivalent impedance and the equivalent metafluid for the lossless case, Fig. 3a and c, with almost identical results from the two models for both cells. When viscothermal losses are included, the metafluid model prediction is expected to be close to the narrow region approximation because of the common usage of the complex propagation constant from Pierce’s model. Pierce’s loss modelling is a low-order modelling approximation of the full viscothermal prediction, and some discrepancies between the two are expected. Figure 3c and d show that the metafluid model line is almost superimposed to the narrow region approximation line, while the equivalent impedance model follows the thermoviscous acoustics results. The differences between the impedance and the metafluid model can be ascribed to the slightly different numerical meaning of the complex sound speed in the two models. For the impedance model, it reduces to a change of the boundary conditions of the problem. In contrast, in the metafluid model, the acoustic waves actually propagate in a dissipative medium, preserving the sound-hard boundary condition in the cell’s domain. The same considerations can be made for the SC cells. Figure 4 presents results for a strongly coiled cell (cell #1) and an almost straight one (cell #5). The transmission coefficient predicted from the metafluid model reproduces the lossless numerical simulations almost perfectly, and the solutions from the three different simulation strategies are in very good agreement. The introduction of viscothermal losses in the metafluid model leads to a solution closely related to the narrow region approximation for the SC cells too. Finally, the use of the parameter ${\hat{a}}_{\text {walls}}$ in the metafluid model to control the extent of the region where the losses are relevant is confirmed to be appropriate by the agreement reached for the HR cells between the narrow region approximation and the lossy metafluid models. Results for other cells of both HR and SC type can be found in the Supplementary Information.

**Figure 5:**
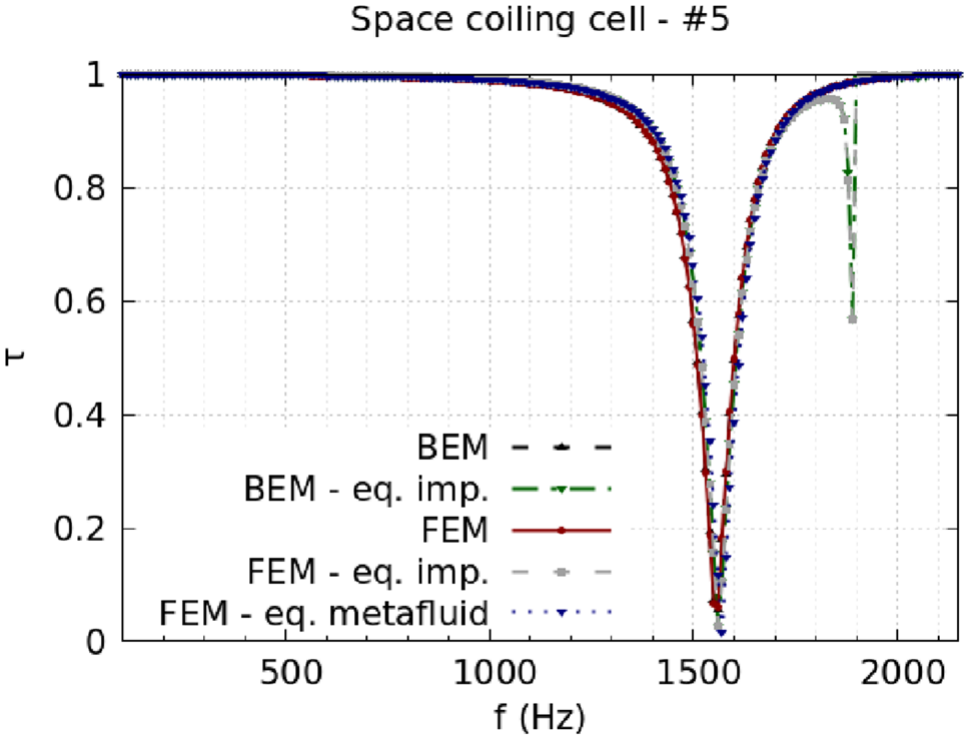
Comparison between FEM and BEM simulations with the complete geometry and using the equivalent impedance model for SC cell #5.

It has been noted that the equivalent impedance model shows a spurious response for some frequencies, more pronounced for those cells with a wider opening at the duct connection (*i.e.* large *d* or $r_n$ values for SC and HR cells, respectively). Oscillations in the $\tau$ spectrum are, in fact, more visible for the SC #5 cell around 1900 Hz but are also there for the HR #7 (slightly visible at around 1700 Hz on the grey line). The transmission coefficient for the SC #5 cell obtained from Boundary Element Method (BEM) simulations using the equivalent impedance model exhibit the very same behavior, as shown in Fig. 5.

**Figure 6:**
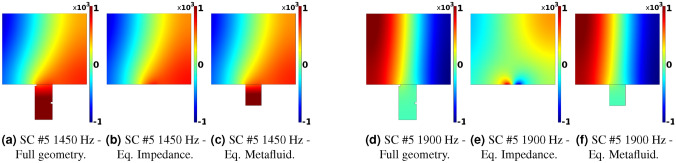
Real part of the acoustic pressure field for the SC cell #5 simulated with the complete geometry, equivalent impedance, and equivalent metacontinuum at 1450Hz (**a**)–(**c**) and 1900Hz (**d**)–(**f**).

The observed response can be attributed to the effect of the impedance wall boundary condition on the modal content of the duct section. If the imaginary part of the impedance is a positive number, the eigensolutions of the modal problem may be found not so far from their hard wall values. However, for a small enough real part of the impedance, the acoustic modal content is deeply affected by the presence of a negative imaginary part (for $+i\omega t$-sign convention) of impedance in the boundary conditions^54^, and the cut-on mode no longer has the shape of a plane wave. The field is characterized by the presence of a so-called surface wave, having its maximum amplitude on the wall and decaying exponentially away from it, affecting the validity of the hypotheses used to calculate the transmission coefficient. In Fig. 6, the real part of the acoustic pressure is visualized for the SC #5 cell at 1450Hz, and 1900 Hz. The figure shows the results obtained imposing a non-reflecting boundary condition for both the inlet and outlet sections, comparing the solutions from the equivalent impedance in (b) and (e), the metacontinuum in (c) and (f), and the complete geometry simulations in (a) and (d). The distortion of the solution for the impedance case at 1900Hz is evident, while the metacontinuum line is in accordance with the reference simulations. The magnitude of the effect is directly correlated with the extension of the impedance patch. At the same time, the presence of a real part in the complex-valued boundary condition partially mitigates the phenomenon, making it almost invisible for the HR #7 cell (whose neck involves a significant effect of the thermoviscous losses). Using the metafluid model allows overcoming this issue, as the boundary conditions involved in the solution are again of the acoustically hard wall type only. No spurious effects are present in the transmission spectra, even for the cells with wider mouths facing the main duct.

Furthermore, the metafluid model showed advantages regarding the required computational time compared to the full thermoviscous simulations and the narrow region approximation. The simulation time required using the metafluid or the boundary impedance model is approximatively the same. The full thermoviscous model obviously requires longer and resource-demanding simulations, as a fine meshing of the acoustic boundary layer in the cells is required for accurate calculations, and more degrees of freedom (DOF) are solved for each mesh node. The simplified geometry used by the metafluid domain also allows for fewer degrees of freedom to be solved than the narrow region approximation model, which, on the contrary, needs the original geometry to be meshed. The converged mesh with quadratic Lagrangian triangular elements used for the lined duct segment problem produced, for the SC cell #1, about 20000 DOF for the narrow region approximation, about 11000 for the metafluid model and about 70000 for the full thermoviscous analysis (the exact number of DOF is dependent on the specific cell; however, the order of magnitude is very similar for all the cells considered). When run on a workstation with an Intel Xeon E7 v4 CPU, this translated in about 8 minutes required for the simulation using the thermoviscous model, about 2 minutes using the narrow region approximation and about 50 seconds for the metafluid model.

## Discussion

This study presented a metacontinuum model for the acoustic modelling of metamaterials and metasurfaces. The equivalence between the original design and the metacontinuum is set through the Transformation Acoustic framework, coupled with information from the reactive part of the acoustic impedance of the cell. A strategy to account for viscous and thermal losses in the sample has been presented, starting from Pierce’s complex-valued speed of sound model and introducing a loss weighting parameter to address changes in the duct equivalent diameter. The steps to obtain the metamaterial properties for a general cell can be summarized as follows (i) the equivalent impedance of the cell is evaluated; (ii) an equivalent straight channel length, generally frequency dependent, is obtained from the imaginary part of the impedance; (iii) a coordinate transformation mapping the equivalent straight channel into the metacontinuum channel domain is set; (iv) the metacontinuum properties are retrieved by means of Transformation Acoustics method; (v) thermoviscous losses inside the cell are modeled as the imaginary part of the speed of sound which is added to the previously evaluated properties The model has been implemented in a FEM solver and numerically validated against standard approaches, such as simulations involving the fully detailed geometry of the metasurface cell or replacing the sample with an equivalent impedance boundary condition. The metafluid model reached a good agreement with the reference simulations in the tested cases, both for the exterior acoustics benchmark and the duct acoustics one. In addition, the lossy metacontinuum proved to be effective in predicting the viscous and thermal effects within the channels of the tested cells, providing results in good agreement with classic approaches.

The derivation of the properties of the metacontinuum for a cell generally involves the preliminary calculation of its equivalent impedance. For this reason, the use of the equivalent impedance as a boundary condition replacing the cell, when spurious effects are not present, and the metacontinuum model share the same order of accuracy. Furthermore, the derivation of the model in terms of equivalent metacontinuum sheds light on alternative designs for PGMs’ cells. Pentamode materials are strongly connected to the properties of a metacontinuum, suggesting that such periodic lattices with tailored microstructure can be exploited to obtain the needed and generally anisotropic properties of the continuum. Modern additive manufacturing techniques should represent the natural option to actually build the required geometries which may result in small and intricate features. The use of such structures would allow for combining the acoustic response with peculiar mechanical properties for the cells.

The model has been tested here on space-coiling and Helmholtz resonating cells. However, the bond between the equivalent acoustic impedance of a sample and its metafluid parameters ensures the method’s applicability to be wide and general, valid in principle for any design of the acoustic device.

## Methods

This section presents the methods used in the numerical analyses conducted to validate the metacontinuum model. All the simulations have been performed implementing the anisotropic density and bulk modulus for the metafluids from Eq. (5) in a commercial finite element method code. Equation 1 and the classic wave equation, expressed for periodic fields in the Fourier domain ($e^{i\omega t}$ convention), are solved in the metafluid domain $\Omega _{\text {mf}}$ and $\Omega _{h}$, respectively:

12 $$\begin{aligned} \begin{aligned} \omega ^2 {\tilde{p}} + c_{\tiny {\textsf {ref}}}^2 \hat{K} \nabla \cdot \left( \textbf{Q}\varvec{\hat{\rho }}^{-1}\varvec{\hat{\rho }}\nabla p \right)&= 0, \quad \textbf{x}\in \Omega _{\text {mf}}\\ \omega ^2 {\tilde{p}} + c_{\tiny {\textsf {ref}}}^2 \nabla ^2 p&= 0, \quad \textbf{x}\in \Omega _{h} \end{aligned} \end{aligned}$$

**Figure 7:**
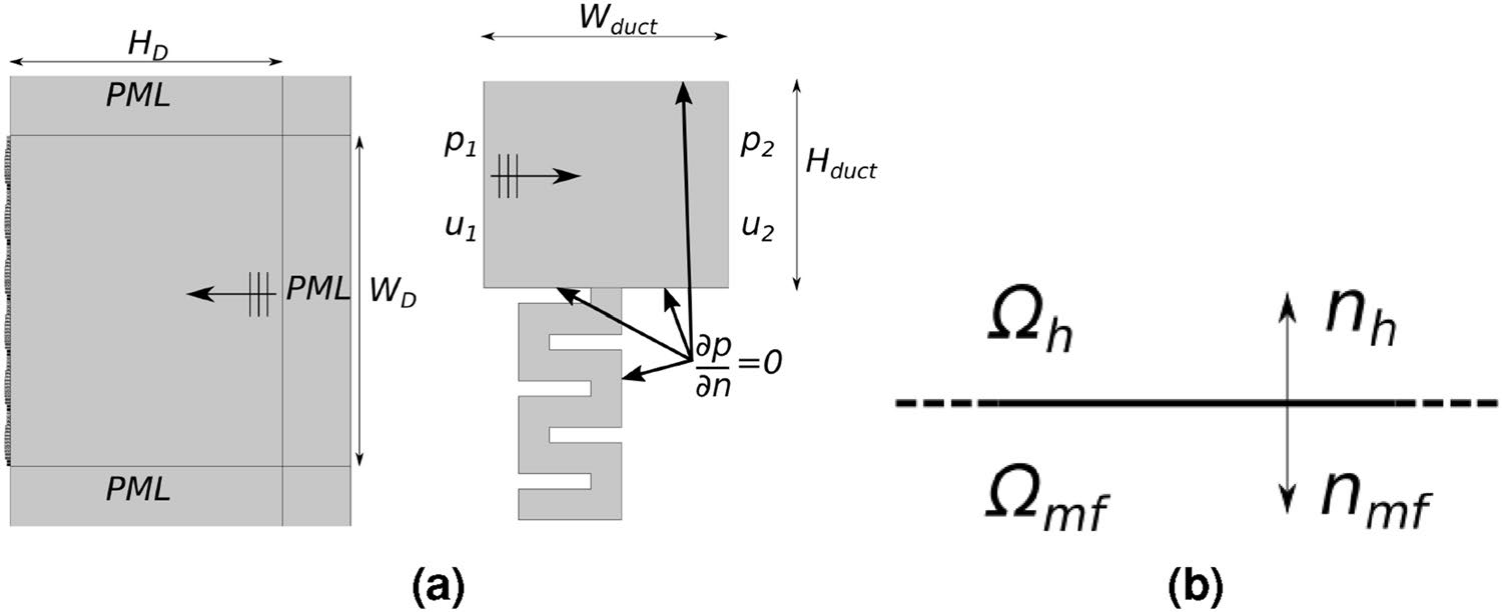
In (**a**): a sketch of the two numerical setups, the extraordinary reflection benchmark for (PGMs), and a lined duct segment for transmission coefficient evaluation. In (**b**): definition of the interface between the metafluid and hosting fluid domains ($\Omega _{\text {mf}} \text { and } \Omega _{h}$) with their respective normal vectors at the interface.

When the complete geometry of a cell is used, a zero normal acceleration boundary condition is imposed at all of its walls, *i.e.* sound hard wall, $\partial p / \partial n = 0$ (see Fig. 7a), and the same holds on the external boundaries of $\Omega _{\text {mf}}$ in the simulations using the equivalent metafluid. The continuity of the pressure and acceleration (and hence velocity) field is ensured at the interface between the metafluid cell and the hosting fluid domain $\Omega _h$, *i.e.*, $p_h=p_{\text {mf}}$ and $\left( \varvec{\rho }^{-1} \nabla p_{\text {mf}} \right) \cdot \textbf{n}= \rho _0^{-1} \nabla p_h \cdot \textbf{n}$, being $\textbf{n}_h = - \textbf{n}_{ms} = \textbf{n}$ (see Fig. 7b). Two numerical setups are investigated, the first involving an exterior acoustics problem of extraordinary reflection from a boundary, the second assessing the transmission loss in a duct segment, both decorated either with Space-Coiling (SC) or Helmholtz Resonator (HR) cells.

The HR and SC cells used have been designed through the optimization procedure described in^38^. Therefore, the design parameters were set to be the original ones from the cited work for the SC, expressed in terms of $a,\,b,\,d,\,t,\,w$ or widths and heights of the necks and cavities for the HR ones, $r_n,\,r_c,\,h_n,\,h_c$ as described in Fig. 1. The values of optimized parameters for the two designs are reported in the Supplementary Information for both SC and HR cells, with a nominal wavelength of $\lambda ^* = 0.19$.

The first case is a classic test for the PGM for reflection. It consists in observing the reflection angle of an incoming planar acoustic perturbation after its interaction with the lined boundary^41,42^. The Generalized Snell’s Law is used to design the phase shift gradient required for the metasurface to attain the desired reflection behavior. Unit cells are then placed accordingly in a periodic pattern. The PGM benchmark simulation includes six repetitions of the metasurface superblock, made of a tailored arrangement of the eight elementary cells. The hosting fluid occupies a domain that extends to all the cells in width, $W_D = n\,w_{\text {cell}}$, with height $H_D = W_D/1.2$, Fig. 7a. For the duct benchmark, only one cell at a time is included in the duct segment, which sizes are $H_{\text {duct}} = 0.08$m and $W_{\text {duct}} = 2\,w_{\text {cell}}$ (with $w_{\text {cell}} = \lambda ^*/8$). Perfectly matched layers are set in the PGM benchmark to avoid undesired reflections from the external boundaries of the computational domain; the duct segment is considered acoustically rigid on its upper and lower boundaries.

The results from the complete geometry and the ones from the equivalent metafluid are then compared qualitatively for the PGM reflection setup; the transmission coefficient $\tau$ is evaluated for the duct setup, also comparing results from the more conventional equivalent impedance modeling approach. The transmission coefficient for all the modeling approaches are calculated by evaluating the four-pole parameters of the transfer matrix of the acoustic system, which links the average acoustic pressures and velocities on the inlet and outlet sections (${\bar{p}}_1$,${\bar{p}}_2$, and ${\bar{u}}_1$, ${\bar{u}}_2$, respectively, the bar meaning the average operation along the section)

13 $$\begin{aligned} \begin{bmatrix} {\bar{p}}_1 \\ \rho _0 c_{\tiny {\text { 0}}}{\bar{u}}_1 \end{bmatrix} = \begin{bmatrix} A \, B \\ C \, D \end{bmatrix} \begin{bmatrix} {\bar{p}}_2 \\ \rho _0 c_{\tiny {\text { 0}}}{\bar{u}}_2 \end{bmatrix} \end{aligned}$$

The numerical evaluation of the four pole parameters can be done in two steps, using two different sets of boundary conditions at the inlet and outlet: A and C are obtained by imposing $u_1=1$ and $u_2=0$; inverting the assignment ($u_1=0$ and $u_2=1$) allows for the evaluation of B and D. The four poles allows for the evaluation of the transmission coefficient $\tau$

14 $$\begin{aligned} A&= \left. \frac{{\bar{p}}_1}{{\bar{p}}_2} \right| _{\begin{matrix} u_1=1 \\ u_2=0\end{matrix}}, \quad B = \left. \frac{\bar{p}_1 - A {\bar{p}}_2}{\rho _0 c_{\tiny {\text { 0}}}} \right| _{\begin{matrix} u_1=0 \\ u_2=1\end{matrix}}, \quad C = \left. \frac{-\rho _0 c_{\tiny {\text { 0}}}}{{\bar{p}}_2} \right| _{\begin{matrix} u_1=1 \\ u_2=0\end{matrix}}, \nonumber \\ \quad D&= \left. \frac{-C \bar{p}_2}{\rho _0 c_{\tiny {\text { 0}}}} \right| _{\begin{matrix} u_1=0 \\ u_2=1\end{matrix}}, \quad \tau = \frac{2}{|A+B+C+D|} \end{aligned}$$

The transmission coefficient spectrum is evaluated from 100 Hz up to 2150 Hz, just below the cut-off frequency of the duct $f<c_{\tiny {\text { 0}}}/2H_{\text {duct}}$, for the three cases of *i)* complete geometry simulation, *ii)* equivalent surface impedance modeling, *iii)* equivalent metacontinuum. The duct acoustic transmission benchmark is also used to address the ability of the equivalent metafluid model to predict the effects of viscosity and thermal conduction correctly. These are included, as already mentioned, in the equivalent metafluid through a complex-valued speed of sound; the same approach has been applied for the equivalent impedance. The same Pierce model is also used for the simulations involving the complete geometry of the cells, being the complex speed of sound considered in the neck portion of the HR cells and over the entire length of the SC channel; this approach is referred to as “narrow regions approximation”. The results from these models are also compared with the complete viscothermal acoustic model, solving the full linearized Navier-Stokes (momentum), continuity, and energy conservation equations, along with the linearized equation of state in the cell domain

15 $$\begin{aligned} i\omega \rho + \nabla \cdot \left( \rho _0 {\textbf{u}} \right)&= 0 \end{aligned}$$

16 $$\begin{aligned} i \omega \rho _0 {\textbf{u}}&= \nabla \cdot \left[ -p\textbf{I}+ \mu \left( \nabla {\textbf{u}} + \left( \nabla {\textbf{u}} \right) ^T \right) - \left( \frac{2}{3} \mu - \mu _B \right) \left( \nabla \cdot {\textbf{u}} \right) \textbf{I}\right] \end{aligned}$$

17 $$\begin{aligned} \rho _0 c_p \left( i\omega T + {\textbf{u}}\cdot \nabla T_0 \right) - \alpha _p T_0 \left( i \omega p + {\textbf{u}} \cdot \nabla p_0 \right)&= \nabla \cdot \left( c_k \nabla T \right) \end{aligned}$$

18 $$\begin{aligned} \rho&= \rho _0\left( \beta _T p - \alpha _p T\right) \end{aligned}$$

where $\beta _T = \frac{1}{\rho _0}\frac{\gamma }{c^2}$ is the isothermal compressiblity and $\alpha _p = \frac{1}{c}\sqrt{\frac{c_p \left( \gamma -1 \right) }{T_0}}$ is the isobaric thermal expansion coefficient.

### Supplementary Information

Supplementary Information.

## Data Availability

All data generated or analysed during this study are included in this published article and its supplementary information files.
