# Supplementary material for: A metacontinuum model for phase gradient metasurfaces
Source: Sci Rep. 2023 Aug 10;13:13038. doi: 10.1038/s41598-023-39956-z (PMC10415379; doi:10.1038/s41598-023-39956-z)
Supplement: Supplementary file 1 — Supplementary Information. [file 41598_2023_39956_MOESM1_ESM.pdf]

Supplementary Information

Design of Metacontinua in the Aeroacoustic Spacetime

Giorgio Palma and Umberto Iemma

Roma Tre University, Department of Civil, Computer Science and Aeronautical Technologies Engineering, Rome, 00146, Italy

Corresponding author e-mail: giorgio.palma@uniroma3.it

A metafluid model for phase gradient metasurfaces

Acoustic impedance model for a Helmholtz resonator

In the main article, an impedance model for the Helmholtz resonator cells is used. We report here some detail on its derivation. In the equation of motion of the mass-spring system, representing the resonator in Fig.S1, the displacement of the mass element y_n can be written as a function of the pressure perturbation on the neck section

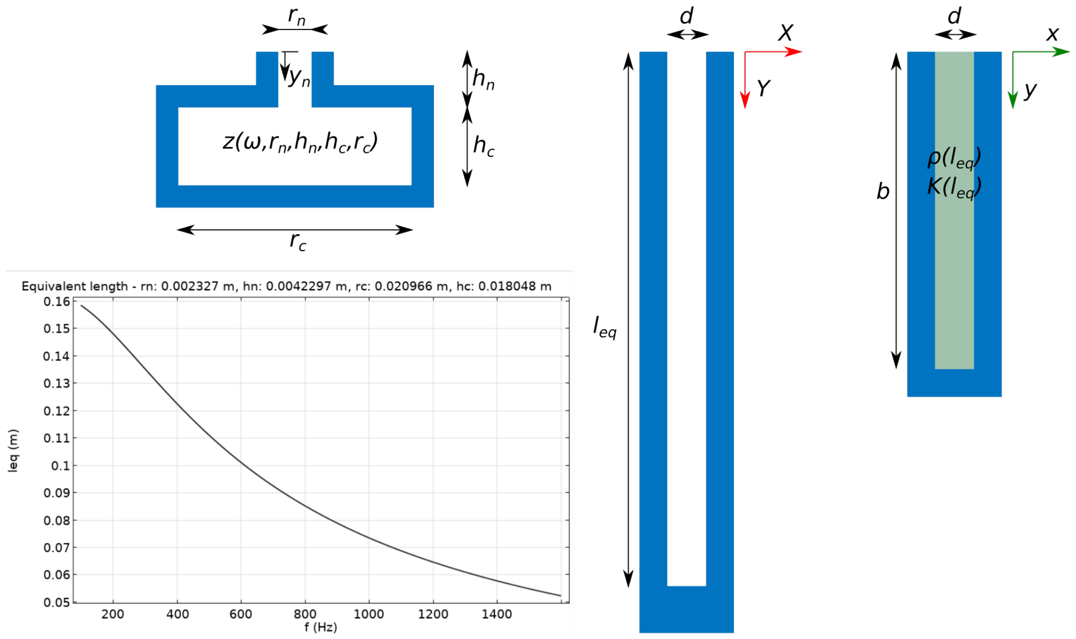

Figure S1. The design of the Helmholtz resonator defines its surface impedance, from which an equivalent length, function of frequency, for a straight duct with the same impedance can be derived. The properties of the metafluid are then evaluated, arbitrarily setting its domain thickness, using the procedure described in the article.

$$m\frac{d^2y_n}{dt^2} = (p+\Delta p)r_n, \qquad m = \rho_0 r_n \hat{h}_n \tag{S1}$$

Within the limit of small variations for the involved variables, we can write

$$m\frac{d^2y_n}{dt^2} = pr_n + \frac{dp}{d\rho}(-\rho_0^2 dv)r_n \qquad d\rho = -\rho_0^2 dv \tag{S2}$$

where $\frac{dp}{d\rho} = c_0$ and $dv = \frac{r_n y_n}{\rho_0 V}$, with $V = h_c r_c$. Hence, substituting in the previous, the governing equation for the displacement y_n becomes

$$m\frac{d^2y_n}{dt^2} + \frac{c_0^2\rho_0 y_n r_n^2}{V} = pr_n \tag{S3}$$

Considering harmonic variations of the quantities $p = \tilde{p}_n e^{i\omega t}$ $y = \tilde{y}_n e^{i\omega t}$, Eq.S3, and dropping the time exponential terms, one obtains

$$-\omega^2 m\tilde{y}_n + \frac{c_0^2\rho_0 r_n^2}{V}\tilde{y}_n = \tilde{p}r_n \tag{S4}$$

Recalling that the acoustic particle velocity is $\tilde{u} = i\omega\tilde{y}_n$, the expression for the specific acoustic impedance is retrieved

$$z = \frac{1}{i\omega}(-\omega^2\rho_0\hat{h}_n + \frac{c_0^2\rho_0 r_n}{h_c r_c}) \tag{S5}$$

The term $\hat{h}_n$ is the length of the neck of the resonator, accounting for the end corrections as described by Bies & Calton for each side of the neck[1,2]

$$\hat{h}_n = h_n + 2\left(0.85 r_n \left(1 - 1.33\frac{r_n}{r_c}\right)\right) \tag{S6}$$

Design variables for PGMS cells

The HR and SC cells used have been designed through the optimization procedure described in[3]. Here, in Tab. S1 and Tab. S2, we report the values obtained for a set of eight cells for each type.

	#1	#2	#3	#4	#5	#6	#7	#8
r_n (mm)	0	15.0	14.819	7.976	7.2855	5.9505	5.0	5.0
r_c (mm)	-	46.5	46.482	45.798	45.729	45.595	45.5	45.5
h_n (mm)	-	3.032	4.2043	3.8733	4.243	4.5	8.0613	22.5
h_c (mm)	-	27.288	37.838	34.859	38.187	40.5	36.939	22.5

Table S1. Optimized cells design parameters for HR-based PGMS metasurface, nominal $\lambda^* = 0.19$m.

	#1	#2	#3	#4	#5	#6	#7	#8
a (mm)	16.447	13.863	15.935	17.276	19.947	19.828	19.632	14.567
b (mm)	23.747	35.985	41.557	41.557	39.762	33.646	23.992	23.747
d (mm)	2.0581	5.2973	8.4895	11.953	17.981	14.923	10.096	9.9737
t (mm)	3.6502	4.9418	3.9057 m	3.2355	1.8998	1.9594	2.0576	4.59
n_w	6	5	4	3	2	2	2	2
w (mm)	1.8998	1.8998	1.8998	1.8998	1.8998	1.8998	1.8998	1.8998

Table S2. Optimized cells design parameters for SC-based PGMS metasurface, nominal $\lambda^* = 0.19$m.

Additional results

Space-coiling cells

We report in Figs. from S2 to S8 some additional results for the space-coiling cells, in terms of transmission coefficient.

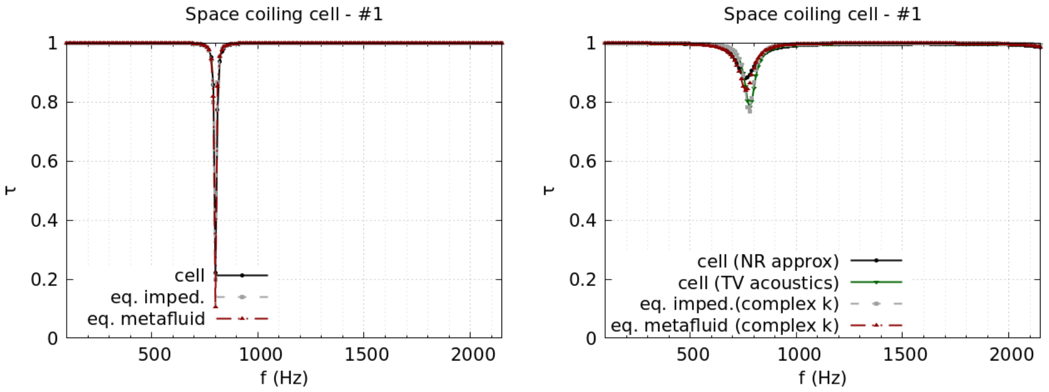

(a) SC cell #1 without losses.

(b) SC cell #1 with losses.

Figure S2. SC cells #1, comparison of transmission coefficient spectra between full geometry, equivalent impedance, and equivalent metafluid lossless simulations (a) and including thermoviscous losses (b). For the lossy case , narrow regions approximation (NR approx) and a full thermoviscous acoustic simulation (TV acoustics) are compared with the equivalent impedance and metafluid modelling with complex wavenumber (complex k).

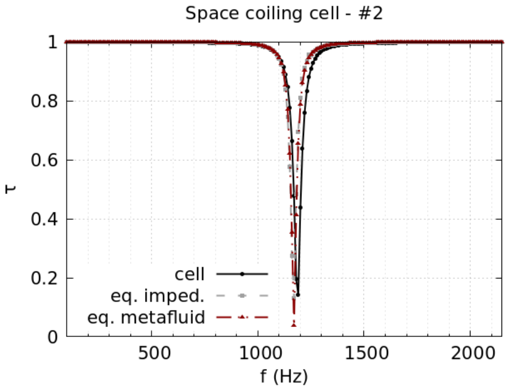

(a) SC cell #2 without losses.

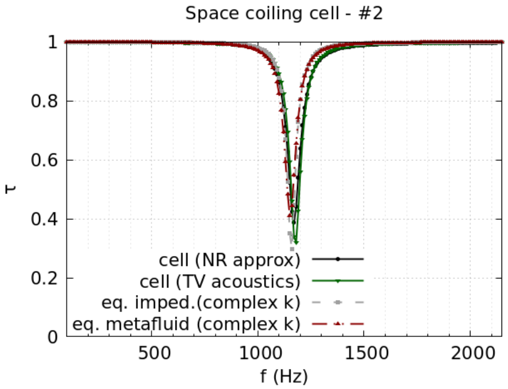

(b) SC cell #2 with losses.

Figure S3. SC cells #2, comparison of transmission coefficient spectra between full geometry, equivalent impedance, and equivalent metafluid lossless simulations (a) and including thermoviscous losses (b). For the lossy case , narrow regions approximation (NR approx) and a full thermoviscous acoustic simulation (TV acoustics) are compared with the equivalent impedance and metafluid modelling with complex wavenumber (complex k).

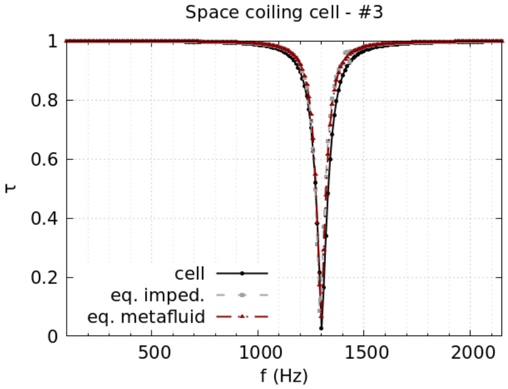

(a) SC cell #3 without losses.

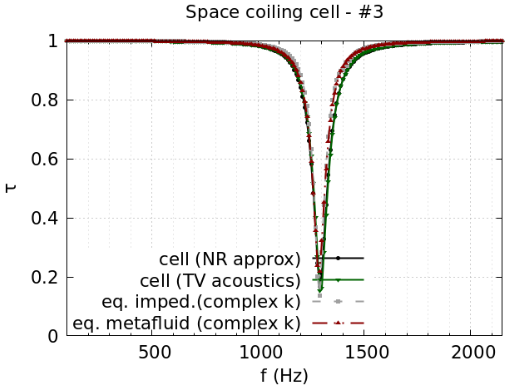

(b) SC cell #3 with losses.

Figure S4. SC cells #3, comparison of transmission coefficient spectra between full geometry, equivalent impedance, and equivalent metafluid lossless simulations (a) and including thermoviscous losses (b). For the lossy case , narrow regions approximation (NR approx) and a full thermoviscous acoustic simulation (TV acoustics) are compared with the equivalent impedance and metafluid modelling with complex wavenumber (complex k).

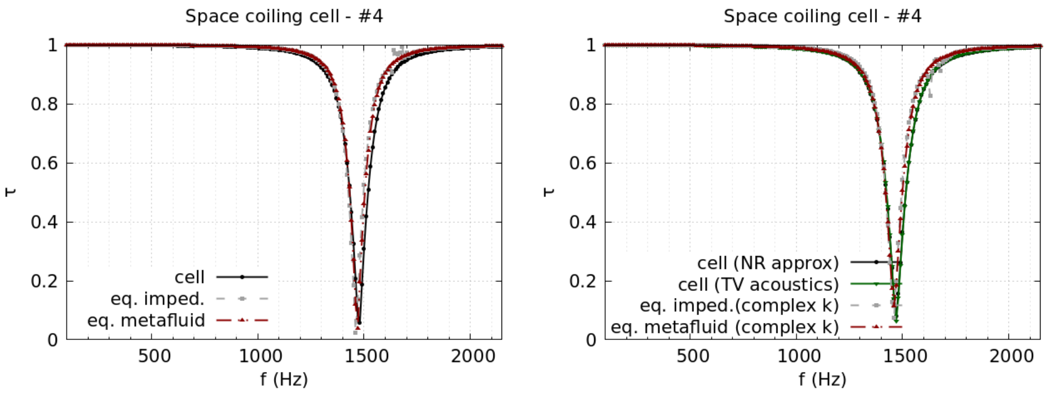

(a) SC cell #4 without losses.

(b) SC cell #4 with losses.

Figure S5. SC cells #4, comparison of transmission coefficient spectra between full geometry, equivalent impedance, and equivalent metafluid lossless simulations (a) and including thermoviscous losses (b). For the lossy case , narrow regions approximation (NR approx) and a full thermoviscous acoustic simulation (TV acoustics) are compared with the equivalent impedance and metafluid modelling with complex wavenumber (complex k).

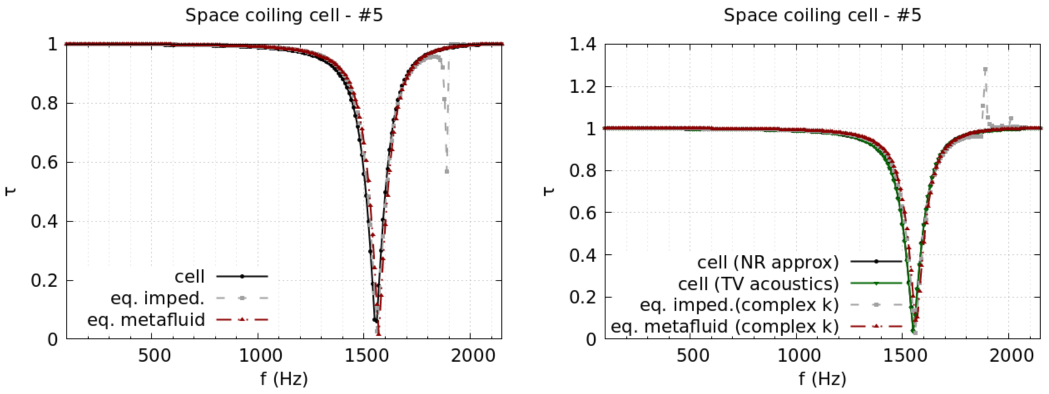

(a) SC cell #5 without losses.

(b) SC cell #5 with losses.

Figure S6. SC cells #5, comparison of transmission coefficient spectra between full geometry, equivalent impedance, and equivalent metafluid lossless simulations (a) and including thermoviscous losses (b). For the lossy case , narrow regions approximation (NR approx) and a full thermoviscous acoustic simulation (TV acoustics) are compared with the equivalent impedance and metafluid modelling with complex wavenumber (complex k).

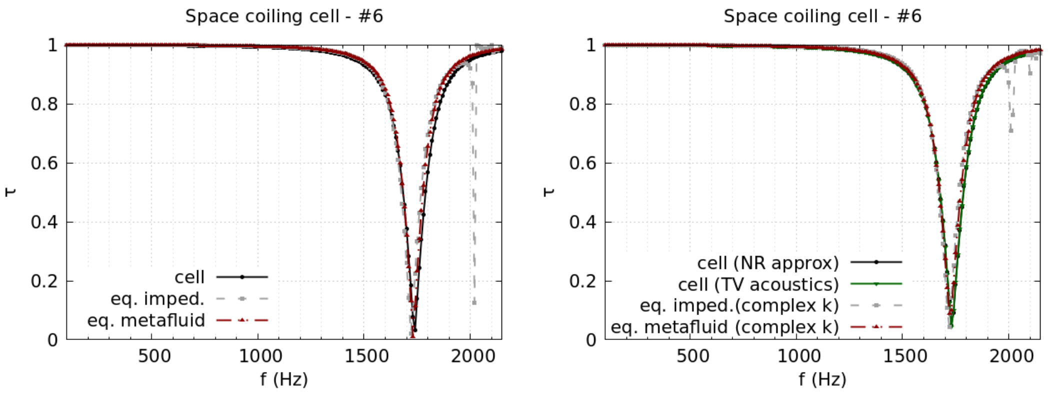

(a) SC cell #6 without losses.

(b) SC cell #6 with losses.

Figure S7. SC cells #6, comparison of transmission coefficient spectra between full geometry, equivalent impedance, and equivalent metafluid lossless simulations (a) and including thermoviscous losses (b). For the lossy case , narrow regions approximation (NR approx) and a full thermoviscous acoustic simulation (TV acoustics) are compared with the equivalent impedance and metafluid modelling with complex wavenumber (complex k).

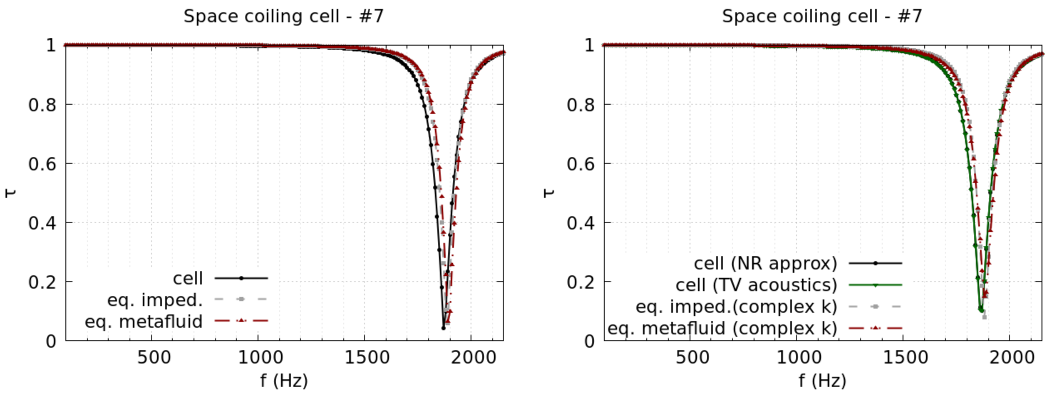

(a) SC cell #7 without losses.

(b) SC cell #7 with losses.

Figure S8. SC cells #7, comparison of transmission coefficient spectra between full geometry, equivalent impedance, and equivalent metafluid lossless simulations (a) and including thermoviscous losses (b). For the lossy case , narrow regions approximation (NR approx) and a full thermoviscous acoustic simulation (TV acoustics) are compared with the equivalent impedance and metafluid modelling with complex wavenumber (complex k).

Helmholtz resonator cells

In Figs. from S9 to S14, we report the results in terms of transmission loss for some of the Helmholtz Resonator cells

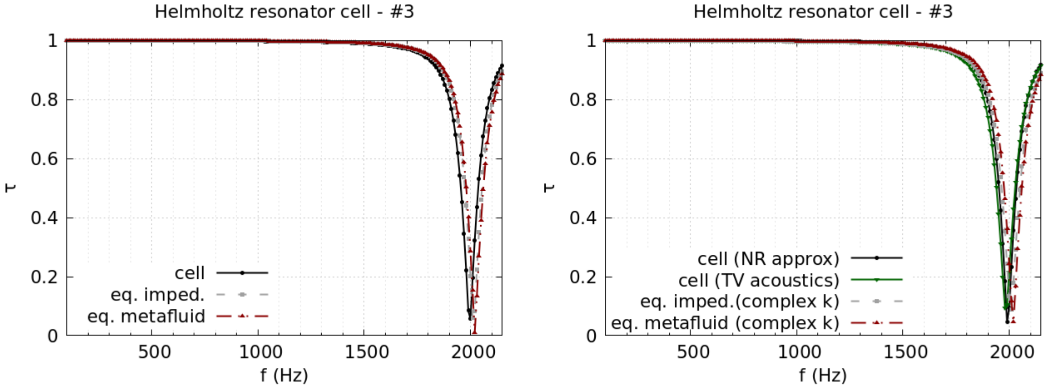

(a) HR cell #3 without losses.

(b) HR cell #3 with losses.

Figure S9. HR cells #3, comparison of transmission coefficient spectra between full geometry, equivalent impedance, and equivalent metafluid lossless simulations (a) and including thermoviscous losses (b). For the lossy case , narrow regions approximation (NR approx) and a full thermoviscous acoustic simulation (TV acoustics) are compared with the equivalent impedance and metafluid modelling with complex wavenumber (complex k).

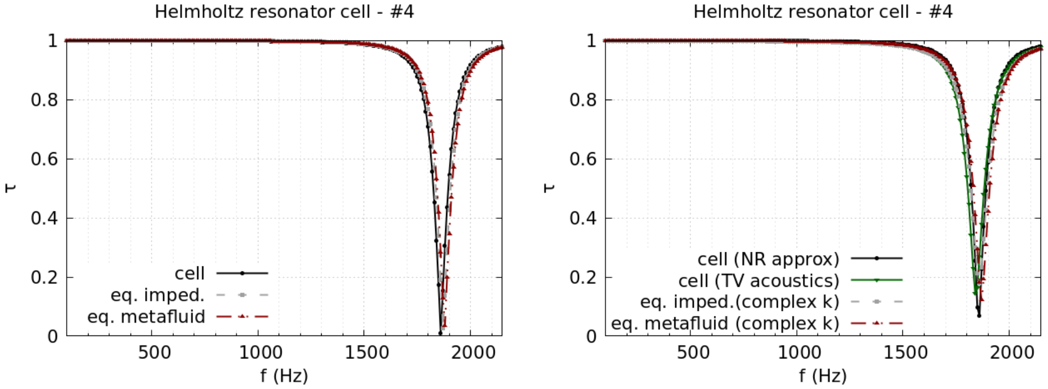

(a) HR cell #4 without losses.

(b) HR cell #4 with losses.

Figure S10. HR cells #4, comparison of transmission coefficient spectra between full geometry, equivalent impedance, and equivalent metafluid lossless simulations (a) and including thermoviscous losses (b). For the lossy case , narrow regions approximation (NR approx) and a full thermoviscous acoustic simulation (TV acoustics) are compared with the equivalent impedance and metafluid modelling with complex wavenumber (complex k).

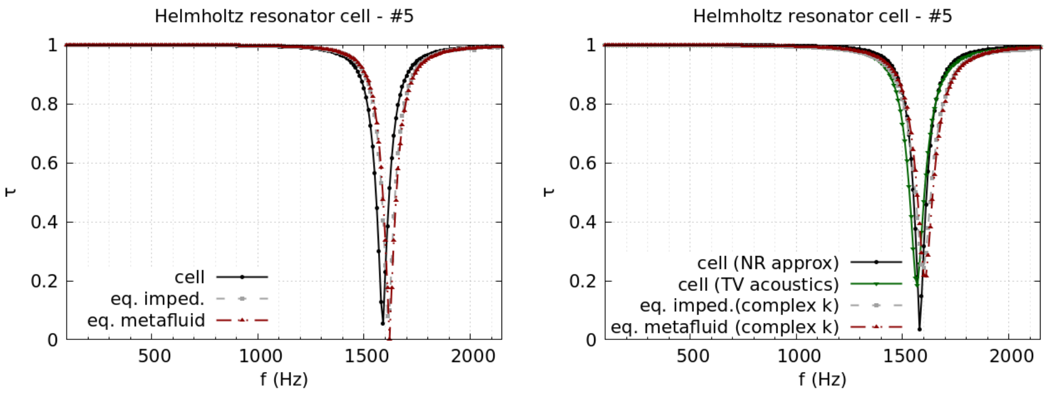

(a) HR cell #5 without losses. **(b)** HR cell #5 with losses.

Figure S11. HR cells #5, comparison of transmission coefficient spectra between full geometry, equivalent impedance, and equivalent metafluid lossless simulations (a) and including thermoviscous losses (b). For the lossy case , narrow regions approximation (NR approx) and a full thermoviscous acoustic simulation (TV acoustics) are compared with the equivalent impedance and metafluid modelling with complex wavenumber (complex k).

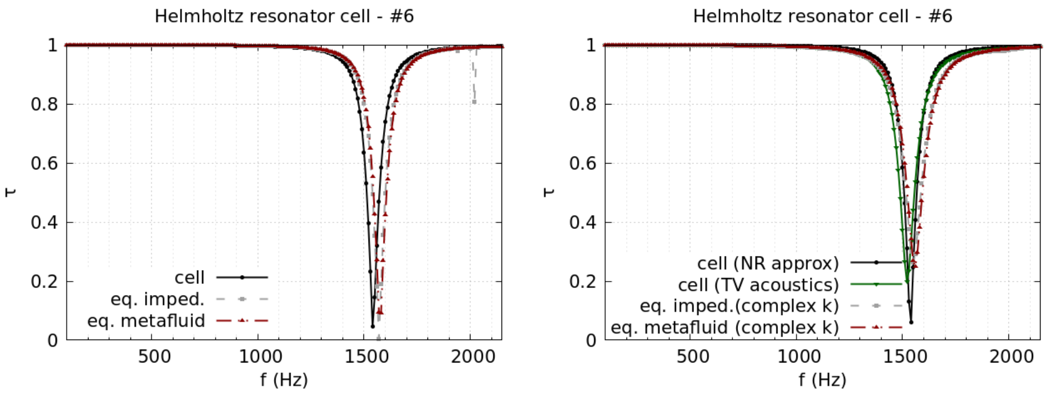

(a) HR cell #6 without losses. **(b)** HR cell #6 with losses.

Figure S12. HR cells #6, comparison of transmission coefficient spectra between full geometry, equivalent impedance, and equivalent metafluid lossless simulations (a) and including thermoviscous losses (b). For the lossy case , narrow regions approximation (NR approx) and a full thermoviscous acoustic simulation (TV acoustics) are compared with the equivalent impedance and metafluid modelling with complex wavenumber (complex k).

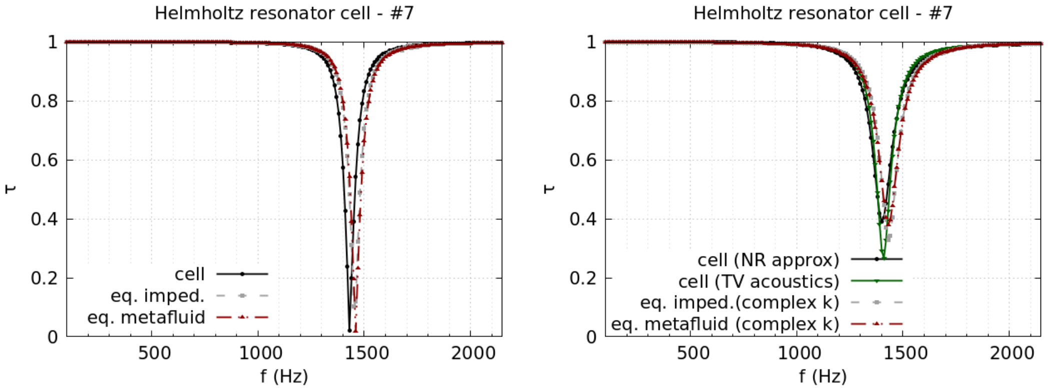

(a) HR cell #7 without losses. **(b)** HR cell #7 with losses.

Figure S13. HR cells #7, comparison of transmission coefficient spectra between full geometry, equivalent impedance, and equivalent metafluid lossless simulations (a) and including thermoviscous losses (b). For the lossy case , narrow regions approximation (NR approx) and a full thermoviscous acoustic simulation (TV acoustics) are compared with the equivalent impedance and metafluid modelling with complex wavenumber (complex k).

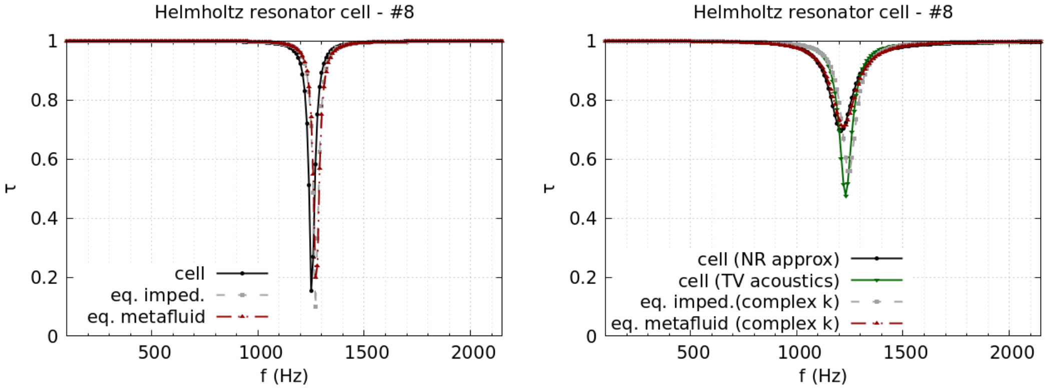

(a) HR cell #8 without losses. **(b)** HR cell #8 with losses.

Figure S14. HR cells #8, comparison of transmission coefficient spectra between full geometry, equivalent impedance, and equivalent metafluid lossless simulations (a) and including thermoviscous losses (b). For the lossy case , narrow regions approximation (NR approx) and a full thermoviscous acoustic simulation (TV acoustics) are compared with the equivalent impedance and metafluid modelling with complex wavenumber (complex k).

References

1. Bies, D. & Hansen, C. *Engineering Noise Control: Theory and Practice, Fourth Edition* (Taylor & Francis, 2009).
2. Calton, M. F. & Sommerfeldt, S. D. Modeling acoustic resonators: From theory to application. In *INTER-NOISE and NOISE-CON Congress and Conference Proceedings*, vol. 250, 3184–3191 (Institute of Noise Control Engineering, 2015).
3. Palma, G., Cioffi, I., Centracchio, F., Burghignoli, L. & Iemma, U. *Steering of Acoustic Reflection from Metasurfaces through Numerical Optimization* (2019).
